# Bridging the Gap Between Diabetes and Cardiovascular Disease: A Comparative Review of Different Glucagon-Like Peptide-1 (GLP-1) Agonists: Efficacy, Safety, and Patient Outcomes

**DOI:** 10.7759/cureus.74345

**Published:** 2024-11-24

**Authors:** Feras A Alghamdi, Hussein A Alshegifi, Reema S Alhuthayli, Turki Helal, Turki A Huwait, Turki Alharbi, Abdulrahman F Akbar, Wejdan Alshehri, Sultan M AlSheikh

**Affiliations:** 1 Family Medicine, King Fahad Military Medical Complex, Jeddah, SAU; 2 Family Medicine, King Abdulaziz Medical City Jeddah, Jeddah, SAU; 3 Medicine, King Abdulaziz University Faculty of Medicine, Jeddah, SAU

**Keywords:** adverse events, diabetes mellitus, glp-1ra, glycemic control, type 2 diabetes

## Abstract

Diabetes mellitus, particularly Type 2 diabetes (T2DM) remains a significant concern globally with an increase in prevalence reported in recent years. If diabetes is not managed properly, it can lead to several complications including an increased risk of cardiovascular disease (CVD). Cardiovascular complications such as coronary heart disease, peripheral artery disease, and stroke are common among individuals with diabetes. Therefore, the timely management of diabetes becomes very important. Glucagon-like peptide-1 receptor agonists (GLP-1 RAs) have emerged as a promising class of medications that offer benefits beyond glycemic control. Among various benefits, GLP-1 RAs can promote pancreatic β-cell proliferation and reduce their apoptosis. They also exert central effects on appetite and energy balance. Furthermore, the weight-lowering potential of GLP-1 RAs has also been documented in literature which can provide indirect benefit to CVD prevention. Long-term GLP-1 RAs generally have superior efficacy over short-term GLP-1 RAs in terms of controlling overnight and fasting plasma glucose levels. However, short-acting GLP-1 RAs, such as exenatide and lixisenatide, maintain their influence on gastric emptying during prolonged use. Adverse events, particularly, gastrointestinal adverse events, remain a concern with GLP-1 RA use. These symptoms usually appear at the start of treatment but fade as the body adjusts to the medication. GLP-1 RAs have shown beneficial effects on cardiovascular health, including a reduction in the incidence of major adverse cardiovascular events. In conclusion, GLP-1 RAs provide multifaceted benefits in T2DM as they not only maintain glycemic control but also decrease cardiovascular risk.

## Introduction and background

Background on diabetes and cardiovascular disease

Diabetes is characterized by chronic hyperglycemia, resulting from either insufficient insulin production or the body's resistance to insulin's effects [1,2]. This condition is one of the leading causes of global morbidity and mortality. There are two main types of diabetes, including Type 1 diabetes mellitus (T1DM) and type 2 diabetes mellitus (T2DM). T2DM is the most prevalent form that accounts for nearly 90% of all diabetes cases, whereas T1DM constitutes only 5% to 10% of diabetic cases [2]. According to the World Health Organization (WHO), the burden of diabetes rose significantly from 1980 to 2014, with 108 million affected individuals in 1980 and 422 million in 2014. This rise is more pronounced in low- and middle-income countries compared to high-income countries. From 2000 to 2019, an increase of 3% was reported in the mortality due to diabetes by age [3]. The rising incidence of diabetes is primarily driven by unhealthy lifestyles, aging populations, and increasing obesity rates [4]. Diagnosis typically involves a combination of clinical history, including symptoms and family background, and tests such as hemoglobin A1C (HbA1C) or plasma glucose levels measured during fasting or two hours after glucose intake [2]. A significant portion of the health burden associated with diabetes stems from its macrovascular and microvascular complications. Microvascular complications include nephropathy, retinopathy, and neuropathy, with retinopathy being the most common. Beyond these severe and disabling complications, cardiovascular disease (CVD) remains the leading cause of morbidity and mortality in individuals with T2DM [4].

Diabetes-related cardiovascular complications

Patients with diabetes are particularly at risk of arteriosclerotic cardiovascular diseases (ASCVDs), including coronary heart disease (CHD), peripheral artery disease (PAD), and stroke [4]. A recent systematic review of 4,549,481 individuals with T2DM revealed an overall prevalence of macrovascular complications of 32.2%, with CHD being the most frequently reported form of CVD at 21.2% [5]. Several follow-up studies have reached a similar conclusion, identifying T2DM as a CHD equivalent in a way [6,7]. This is because individuals with T2DM, even without prior signs of CHD, face a risk of developing CHD that is comparable to or even greater than that of those with a history of CHD, particularly among women [6]. In individuals with diabetes, CHD is generally identified at more advanced stages compared to the general population due to the potential for 'silent' ischemia, where symptoms are not apparent. Research has shown that even in those with diabetes who do not have clinically diagnosed CHD, there is evidence of coronary atherosclerosis. This indicates that the process of arteriosclerosis tends to develop more rapidly, earlier, and more extensively in people with diabetes [8].

PAD in individuals with diabetes typically affects the more distal segments of blood vessels and often presents with symptoms such as claudication. It can progress to more severe outcomes, including lower extremity amputation [9]. A cohort study of 1,921,260 individuals, with 34,198 (1·8%) having T2DM reported that PAD was the initial symptom reported in T2DM patients (16.2%), followed by heart failure (14.1%) [10]. Women are more likely to have PAD compared to men [11]. Diabetes-related strokes are primarily caused by diseases affecting the extracranial carotid arteries and both large and small intracranial vessels. The clinical manifestations range from asymptomatic to devastating events like ischemic attacks and both hemorrhagic and ischemic strokes. Diabetes is a significant independent risk factor for stroke, with an incidence 2.5 to 3.5 times higher than that of non-diabetic individuals. After CHD, stroke is the leading cause of death in patients with T2DM [4]. Given the heavy burden diabetes places on healthcare systems, particularly as a driver of CVD, there is an urgent need for improved monitoring, control strategies, and a better understanding of its complications [4]. 

Coronary artery disease (CAD) significantly affects the long-term outlook of patients with diabetes mellitus (DM). It is the leading cause of death in individuals with both type 1 and type 2 DM, and diabetes is linked to a two- to four-fold increase in mortality risk from cardiovascular conditions [12]. More than 70% of people over the age of 65 with DM are likely to succumb to heart disease or stroke. Additionally, patients with DM have a higher risk of death following a myocardial infarction (MI) and generally face a poorer long-term prognosis with CAD [13]. A retrospective study by Booth et al. showed that patients with DM have a CVD almost 15 years before those without DM [14]. Some studies have suggested that females are more affected by DM than males regarding CVD. For example, a study by Ballotari et al. reported that females have higher mortality with CVD associated with diabetes compared to males [15].

Research demonstrates a linear and significant association between fasting plasma glucose levels and CVD risk, regardless of glucose concentration. Dysglycemia - abnormal blood sugar levels - is a particularly strong risk factor for CVD. These findings suggest that treating dysglycemia as a continuous risk factor, similar to blood cholesterol and blood pressure management, could be a more effective strategy for cardiovascular risk assessment and prevention than focusing solely on specific glucose thresholds [4]. The link between diabetes and CVD is underpinned by various pathophysiological mechanisms. While hyperglycemia directly damages endothelial function and promotes atherosclerosis, other factors such as hyperinsulinemia, insulin resistance, and dyslipidemia also contribute to the increased cardiovascular risk in individuals with diabetes [4].

## Review

Mechanism of action

Following an oral glucose load, the incretin effect of GLP-1 and glucose-dependent insulinotropic polypeptide (GIP) - two incretin hormones inactivated by dipeptidyl peptidase 4 (DPP-4) - stimulates insulin production [16,17]. This process may become attenuated or eliminated in T2DM; however, pharmaceutical GLP-1 doses can restore insulin secretion. Benefits of this therapy for T2DM include delayed gastric emptying and, during hyperglycemia, inhibition of pancreatic α-cell glucagon production. Furthermore, GLP-1 receptor agonists can promote pancreatic β-cell proliferation and reduce their apoptosis [18-20]. These drugs mimic the action of endogenous GLP-1, an incretin hormone produced in the intestinal L-cells in response to nutrient ingestion. The mechanism of action of GLP-1 receptor agonists (GLP-1 RAs) is primarily mediated through the activation of GLP-1 receptors (GLP-1 Rs), which are G protein-coupled receptors found in multiple tissues including the pancreas, gastrointestinal (GI) tract, brain, heart, and adipose tissue. By binding to these receptors, GLP-1 RAs exert a range of effects that collectively improve glycemic control [21].

At the pancreatic level, the primary mechanism of action of GLP-1 RA is the potentiation of glucose-dependent insulin secretion from pancreatic beta-cells. When glucose levels rise, such as after a meal, GLP-1 RAs enhance insulin release by increasing intracellular cyclic adenosine monophosphate (cAMP) levels and activating protein kinase A (PKA). This signaling cascade facilitates the opening of voltage-dependent calcium channels and results in calcium influx and subsequent exocytosis of insulin granules [22]. Unlike other secretagogues, such as sulfonylureas, GLP-1 RA only stimulates insulin secretion in the presence of elevated glucose levels, thereby minimizing the risk of hypoglycemia. Additionally, GLP-1 receptor activation inhibits glucagon secretion from pancreatic alpha cells, reducing hepatic glucose production, particularly in the postprandial state. This dual action on insulin and glucagon secretion helps maintain glucose homeostasis [23]. 

GLP-1 also exhibits neuroprotective properties, enhances muscle glucose uptake, reduces hepatic glucose production, and increases satiety through direct hypothalamic effects. Beyond their effects on pancreatic function and the gastrointestinal system, GLP-1 RAs exert central effects on appetite and energy balance [24]. GLP-1 receptors are expressed in several regions of the brain, including the hypothalamus and brainstem, which are critical centers for appetite regulation. When GLP-1 RAs cross the blood-brain barrier, they activate GLP-1 receptors in these areas, leading to the activation of anorexigenic pathways and inhibition of orexigenic pathways [25]. This results in reduced appetite and decreased food intake, contributing to their weight loss effect. The activation of GLP-1 receptors in the brain also affects reward-related eating behavior and helps reduce cravings for high-calorie foods. Moreover, GLP-1 RAs may increase energy expenditure through thermogenesis, although this effect is less pronounced than their impact on appetite suppression [26]. 

GLP-1 RAs lower total cholesterol, and systolic and diastolic blood pressure, and promote an average weight loss of 2.9 kg compared to placebo. Cardiovascularly, GLP-1 RA can reduce infarction extent and overall cardiovascular event risks while enhancing cardiac output, myocardial contractility, coronary blood flow, left ventricular ejection fraction, and endothelial function [27,28]. In T2DM patients, GLP-1 RAs have demonstrated a 1% decrease in glycated hemoglobin (HbA1c) and reduced all-cause mortality compared to control groups [29,30]. The American Diabetes Association asserts that more potent therapies have a higher likelihood of achieving glycemic targets. Specifically, injectable combination therapy, oral combination therapy, high-dose dulaglutide and semaglutide, tripeptide - a combination of gastric inhibitory peptide (GIP) and GLP-1RA - and insulin therapy are all considered highly effective in lowering blood glucose levels [31].

Pharmacokinetics

Absorption

Subcutaneous administration of GLP-1 RA such as exenatide, liraglutide, and semaglutide ensures rapid absorption, reaching peak concentrations within hours. Semaglutide's innovative oral formulation considers bioavailability in its design for gastrointestinal absorption. Effective dosing regimens for oral semaglutide depend on factors like first-pass metabolism and potential interactions with food and other drugs [32].

Distribution

GLP-1 RAs, including semaglutide, liraglutide, and exenatide, have a modest volume of distribution after absorption and primarily remain in the bloodstream. These drugs specifically target GLP-1 receptors in various glucose-regulating tissues, with a particular affinity for pancreatic cells and other metabolic control sites [32].

Metabolism

Exenatide undergoes hydrolysis in the liver and kidneys as its primary metabolism, producing smaller, inactive peptides that are then eliminated through the kidneys. Liraglutide, similar to large protein metabolism, involves proteolytic cleavage in various organs. Neutral endopeptidase (NEP) and dipeptidyl peptidase-4 (DPP-4) are two potentially implicated enzymes that generate smaller, physiologically inactive fragments for subsequent elimination. Serum and tissue proteases facilitate the metabolic breakdown of the polypeptide Semaglutide into individual amino acids [33,34].

Excretion

GLP-1 RAs, such as exenatide, liraglutide, and semaglutide, are primarily cleared via renal elimination. The kidneys play a crucial role in the body's process of eliminating these substances. The rate of renal excretion affects the duration of action and dosing frequency. Compared to short-acting formulations, Semaglutide has a notably longer half-life due to its extended-release (ER) profile [32].

Importance of GLP-1 agonists in treatment and commonly used GLP-1 agonists

GLP-1 RAs are administered through various dosing regimens: exenatide is injected twice daily, lixisenatide and liraglutide are taken once daily, and options like exenatide (once weekly), dulaglutide, albiglutide, and semaglutide are available for weekly administration. A newly approved oral formulation of semaglutide has shown clinical efficacy comparable to its once-weekly subcutaneous counterpart. All GLP-1 RAs function similarly by enhancing insulin secretion in response to hyperglycemia, inhibiting glucagon secretion under hyperglycemic or euglycemic conditions, slowing gastric emptying to mitigate postprandial glucose spikes, and promoting weight loss by reducing calorie intake [35,36]. Short-acting GLP-1 RAs, such as exenatide and lixisenatide, are less effective at controlling overnight and fasting plasma glucose levels but maintain their influence on gastric emptying during prolonged use. In contrast, long-acting GLP-1 RAs, including liraglutide, weekly exenatide, dulaglutide, albiglutide, and semaglutide, exhibit more significant effects on overnight and fasting plasma glucose levels, as well as HbA1c, especially when combined with other oral glucose-lowering medications or basal insulin. However, the effect of these agents on gastric emptying diminishes over time, a phenomenon known as tachyphylaxis [37].

Given their superior or comparable efficacy in reducing HbA1c, coupled with additional benefits such as weight loss and a low risk of hypoglycemia, GLP-1 RAs are increasingly recommended as the preferred first-line injectable therapy for T2DM, often even before insulin. They can also be effectively combined with basal insulin, either as separate or fixed-dose combinations. Among the newer agents, semaglutide stands out for its potent glucose-lowering and weight-reduction effects [38]. Since 2016, several cardiovascular outcome trials have demonstrated the ability of GLP-1 RAs to significantly reduce the risk of cardiovascular events, such as heart attacks and strokes, and associated mortality, particularly in patients with existing atherosclerotic cardiovascular disease (CVD). Although the evidence is less robust in lower-risk populations, treatment guidelines still advocate for the use of GLP-1 RAs in patients with prior cardiovascular events. When choosing between GLP-1 RAs and sodium-glucose cotransporter-2 (SGLT-2) inhibitors - another class that reduces cardiovascular events by primarily mitigating heart failure - the patient's individual risk profile for ischemic versus heart failure complications should guide therapy decisions [39].

GLP-1 RAs may also offer protective benefits against the development of renal complications in type 2 diabetes. Ongoing research in this field is focused on identifying subgroups of patients with type 2 diabetes who derive the greatest benefit from GLP-1 RA therapy, using approaches such as pharmacogenomics and investigating non-responders. Additionally, new potential applications for GLP-1 RAs beyond type 2 diabetes, including T1DM, neurodegenerative disorders, and psoriasis, are under exploration. Since their introduction approximately 15 years ago, GLP-1 RAs have solidified their role as a key class of glucose-lowering agents, with continued potential for expansion in treating T2DM and possibly other diseases [40]. A meta-analysis by Chen et al. reported that GLP-1 RAs have different abilities to control glycemic levels in diabetic patients. They further showed that semaglutide 2.0 mg has the highest efficacy and safety compared to other GLP-1 RAs. A pairwise comparison showed that once-a-week GLP-1 RAs are much better in lowering HbA1c compared to the placebo group [41].

Comparison of different GLP-1 agonists in terms of impact on blood glucose levels and HbA1c

Multiple studies have compared the effects of different GLP-1 agonists on HbA1c and blood glucose levels [42-44]. A comprehensive meta-analysis evaluated the safety and efficacy of 15 GLP-1 RAs, including newer medications like orforglipron, retatrutide, and cagrisema, in individuals with T2DM. All 15 drugs successfully reduced fasting blood glucose levels and HbA1c compared to placebo. Tirzepatide emerged as the most potent GLP-1 RA for lowering both fasting blood glucose and HbA1c, with substantial evidence supporting this finding. Following tirzepatide in order of effectiveness were mazdutide, cagrisema, semaglutide, dulaglutide, and liraglutide [42]. In a direct comparison between tirzepatide and semaglutide, all doses of tirzepatide (5 mg, 10 mg, or 15 mg) resulted in greater reductions in glycated hemoglobin levels than the 1 mg dose of semaglutide [43].

Robust exposure-response time-course models were used to visualize the potential benefits of switching from GLP-1 RA treatment to tirzepatide (5 mg, 10 mg, or 15 mg weekly). These simulations suggest that switching to tirzepatide from approved maintenance doses of Semaglutide or dulaglutide may offer additional glycemic control and weight loss benefits [45]. A study comparing semaglutide and dulaglutide found that semaglutide led to significantly greater reductions in postprandial blood glucose levels. This finding, combined with the more substantial decrease in fasting plasma glucose observed with semaglutide, likely contributes to the notable reductions in HbA1c seen with semaglutide compared to dulaglutide [46]. In a separate study comparing dulaglutide and liraglutide, both drugs showed significant reductions in HbA1c from baseline. The HbA1c reduction with dulaglutide was comparable to that achieved with liraglutide, with similar patterns of reduction over time [47].

Comparison of safety profiles among different GLP-1 agonists (side effects and safety data)

The safety of GLP-1 RAs remains a concern despite their established efficacy in managing glycemic levels in DM patients. Different GLP-1 RAs vary in their clinical efficacy, pharmacokinetic profile, and molecular structure, which can affect their overall safety profile [30,35,36]. 

Common Side Effects of GLP-1 RAs

The most frequently reported side effects of GLP-1 RAs are gastrointestinal in nature, such as nausea, vomiting, and diarrhea. These side effects are dose-dependent and more common when initiating the drug. For example, exenatide and liraglutide are associated with a higher incidence of gastrointestinal side effects compared to longer-acting agents like dulaglutide and semaglutide. A meta-analysis by Monami et al. found that shorter-acting GLP-1 RAs like exenatide are more likely to cause nausea and vomiting compared to longer-acting agents, such as liraglutide and dulaglutide [28]. However, these side effects often diminish over time as patients' bodies adjust to the medication [35,36]. Nausea is the most common side effect that can be reported in approximately 50% of patients [48]. The commonality of nausea has been attributed to delayed gastric emptying and the peak effects of GLP-1 with short-acting formulations [49]. Gastrointestinal side effects, such as constipation, may persist even though they generally lessen over time, and they tend to be less frequent with long-acting GLP-1 RAs [50]. 

SUSTAIN, a phase 3 trial, reported that 20% of patients who took 0.5 mg semaglutide and 24% who took 1 mg semaglutide developed nausea. Furthermore, diarrhea was reported in 13% of patients who received 0.5 mg semaglutide, and among those who took 1 mg semaglutide, 11% had diarrhea [51]. Similarly, Rodbard et al., in their trial reported that nausea occurred in 11.4% of patients receiving semaglutide at a dose of 0.5 mg and in 16.8% of those receiving 1.0 mg, compared to 4.5% of patients on a placebo. Overall, the rate of nausea associated with semaglutide treatment remained around 3% to 5% throughout the duration of the study [52]. Another study reported that gastrointestinal adverse events were much higher in the semaglutide group compared to placebo (37.3% vs. 13.2%) [53]. In a phase 2 clinical trial, the overall incidence of gastrointestinal disturbances was comparable between oral and subcutaneous forms of semaglutide, occurring in 56% and 54% of participants, respectively. Nausea was reported in 34% of those receiving the oral form and 32% of those receiving the subcutaneous form. Vomiting was observed in 16% of the oral group compared to 9% in the subcutaneous group, while diarrhea occurred in 20% of the oral group and 14% of the subcutaneous group. The frequency of GI side effects tends to increase with higher doses of semaglutide, prompting the use of a dose-escalation strategy that begins with a lower initial dose [54].

Comparison of Safety Profiles

The safety profile of GLP-1 RAs can vary significantly based on their formulation and dosing frequency. Longer-acting GLP-1 RAs, such as dulaglutide and semaglutide, are associated with a lower incidence of gastrointestinal side effects [55,56]. This is potentially due to the more gradual and sustained elevation in GLP-1 levels, reducing peak concentrations that may provoke gastrointestinal symptoms. Moreover, the risk of hypoglycemia is generally low with GLP-1 RAs when used as monotherapy or in combination with metformin [57]. However, when combined with sulfonylureas or insulin, the risk can increase [58]. The risk of hypoglycemia also varies among different GLP-1 RAs; for example, lixisenatide has a slightly higher association with hypoglycemia compared to liraglutide and semaglutide, especially in combination therapies [59].

Cardiovascular Safety

Another important aspect of the safety profile of GLP-1 RAs is their cardiovascular safety. Large-scale cardiovascular outcome trials have demonstrated that certain GLP-1 RAs, such as liraglutide and semaglutide, not only provide cardiovascular benefits but also reduce the risk of major adverse cardiovascular events in high-risk patients with T2DM. However, the cardiovascular effects can vary among the GLP-1 RAs, with some agents like lixisenatide showing neutral effects on cardiovascular outcomes [36,60,61]

Pancreatic and Thyroid Safety Concerns

Concerns regarding the long-term safety of GLP-1 RAs, particularly the risk of pancreatitis and thyroid C-cell tumors, have been raised. Some studies have suggested an association between GLP-1 RA use and acute pancreatitis; however, large observational studies and meta-analyses have not consistently confirmed this risk. The FDA has issued warnings regarding the potential risk of thyroid C-cell tumors with long-acting agents like liraglutide and semaglutide based on rodent studies, but no causal relationship has been established in humans [36,60]. 

Patient outcomes and impact on weight management

Long-acting GLP-1 RAs such as liraglutide, semaglutide, exenatide, and dulaglutide have significant effects on reducing fasting and overnight plasma glucose levels and HbA1c [35,36]. However, they also provide cardioprotective effects and reduce the weight of the patients [62]. Furthermore, they improve lipid profiles by decreasing low-density lipoprotein (LDL), total cholesterol, and triglycerides, as demonstrated in a meta-analysis of 35 clinical trials. These drugs also have an anti-inflammatory effect, reducing inflammatory markers and plaque formation in blood vessels [62]. GLP-1 RAs primarily reduce weight by influencing the gastrointestinal system [63]. They slow down gastric emptying, decrease appetite, enhance fullness, and reduce food intake. This weight loss also enhances insulin sensitivity in diabetic patients [62].

A narrative review found that GLP-1 RAs when combined with lifestyle interventions, led to an average weight loss difference of 4% to 6.2% in patients with diabetes, whereas in those without diabetes, the weight loss ranged from 6.1% to 17.4%. Additionally, semaglutide was associated with a more significant weight reduction than liraglutide [64]. In another study, GLP-1 RAs markedly enhanced the chances of attaining at least 5% and 10% weight loss compared to placebo, with relative risks (RR) of 2.51 and 4.11, respectively. Among these, semaglutide demonstrated the greatest effectiveness by achieving a ≥10% weight loss, with an RR of 5.42, followed by tirzepatide, with an RR of 4.29, and liraglutide, with an RR of 2.91 [65].

Patient outcomes and quality of life

T2DM is a prevalent chronic disease that significantly impacts patients' quality of life and healthcare costs, causing a high global burden. CVD is not only one of the most common complications of DM but also a significant contributor to mortality [66]. Global meta-analytic data shows that T2DM reduces life expectancy by 10 years and increases CVD-related mortality compared to individuals without T2DM [67]. Recent guidelines recommend GLP-1 RAs and SGLT2i as preferred treatment options, either as add-on therapy to metformin or as first-line treatment. Currently, seven GLP-1 RA agents are available for treating T2DM patients. Given that CVD is a leading cause of death in T2DM patients, eight trials have assessed the effect of GLP-1 RAs on cardiovascular risk, each focusing on a different GLP-1 RA medication [61,68-72].

Eight trials evaluated subcutaneous GLP-1: ELIXA (lixisenatide) [72], LEADER (liraglutide) [61], SUSTAIN 6 (semaglutide) [68], EXSCEL (exenatide extended-release) [69], Harmony Outcomes (albiglutide) [70], REWIND (dulaglutide) [71], PIONEER 6 (Semaglutide) [39], and AMPLITUDE-O (efpeglenatide) [73] (Table 1). All trials used composite major adverse cardiovascular events - comprising cardiovascular death, nonfatal MI, and nonfatal stroke - as the primary endpoint. These trials demonstrated that GLP-1 did not increase major adverse cardiac events risk in T2DM patients with established CVD or cardiovascular risk factors [67]. Notably, three GLP-1 agents - liraglutide, semaglutide, and dulaglutide - showed a decrease in major adverse cardiac events incidence compared to placebo when added to standard care, highlighting their cardioprotective properties. An additional benefit observed in these trials was GLP-1's ability to reduce albuminuria or urinary albumin-to-creatinine ratio (UACR), which are important markers of progressive renal impairment [67].

**Table 1:** Cardiovascular Outcome Trials for GLP-1 RA T2DM: Type 2 Diabetes Mellitus; MACE: Major Adverse Cardiovascular Events; CI: Confidence Interval; CVD: Cardiovascular Disease; HbA1c: glycated hemoglobin; GLP-1 RA: Glucagon-like peptide-1 receptor agonists

Trial name	Agent	Participants	Patient characteristics	Intervention	Follow up (years)	HbA1c change (%)	Effect on Heart Rate (bpm)	Weight change (kg)	MACE	CVD related findings
ELIXA [72]	Lixisenatide	6068	T2DM, with myocardial infarction or hospitalization due to angina in last 180 days; Age ≥ 30	Lixisenatide (10–20 μg) or placebo	2.1	-0.27	+0.4	-0.6	1.02 (95% CI [0.89, 1.17]	No significant difference was observed in both groups regarding outcomes.
LEADER [61]	Liraglutide	9340	T2DM, with high cardiovascular risk	Liraglutide (1.8 mg) or placebo	3.8	-0.40	+0.3	-2.3	0.87 (95% CI [0.78, 0.97]	Significant difference in first occurrence of death due cardiovascular cause in intervention group compared to control.
SUSTAIN 6 [68]	Semaglutide	3297	T2DM + high cardiovascular risk	Semaglutide (0.5 or 1.0 mg) or placebo	2	-1.1 (on 0.5 mg) -1.4 (on 1.0 mg)	+2.1 (on 0.5 mg) +2.4 (on 1.0 mg)	-3.6 (on 0.5 mg) -4.9 (on 1.0 mg)	0.74 (95% CI [0.58, 0.95]	Significant reduction in the risk of stroke and nonfatal myocardial infarction in intervention group.
EXSCEL [69]	Exenatide extended-release	14,752	T2DM with or without history of cardiovascular disease	Exenatide (2 mg) or placebo	3.2	-0.7	+2.51	-1.27	0.91 (95% CI [0.83, 1.00]	No significant MACE difference between groups. All cause mortality, however, was lower in intervention group.
Harmony Outcomes [70]	Albiglutide	9463	T2DM with history of cardiovascular disease	Albiglutide (30–50 mg) or placebo	1.5	-0.63 (on 8 mg) -0.52 (on 16 mg)	+1.3	-0.66 (on 8 mg) -0.83 (on 16 mg)	0.78 (95% CI [0.68–0.90]	Reduced MACE and risk of myocardial infarction in intervention group.
REWIND [71]	Dulaglutide	9901	T2DM with history of cardiovascular disease	Dulaglutide (1.5 mg) or placebo	5.4	-0.61	+1.87	-1.46	0.88 (95% CI [0.79–0.99]	Significant reduction in the risk of stroke and MACE
PIONEER 6 [39]	Semaglutide	3183	High cardiovascular risk	Semaglutide (14 mg) or placebo	1.3	-0.7	+4	- 4.2	0.79 (95% CI [0.57, 1.11]	Cardiovascular risk profile was not significantly different between groups.
AMPLITUDE-O [73]	Efpeglenatide	4076	T2DM, with high cardiovascular risk	Efpeglenatide (4 or 6 mg)	1.8	-1.24	+3.9	-2.6	0.73 (95% CI [0.58–0.92]	Decreased risk of cardiovascular events in intervention group

In the ELIXA trial, patients with T2DM who had experienced a myocardial infarction were assigned to receive either lixisenatide or a placebo. The primary composite endpoint included cardiovascular death, myocardial infarction, stroke, or hospitalization for unstable angina. A total of 6068 patients were randomized and followed for a median duration of 25 months. The incidence of the primary endpoint was 13.4% in the lixisenatide group and 13.2% in the placebo group. This result indicated that lixisenatide was not superior to placebo. There were no significant differences between the groups regarding hospitalization for heart failure or mortality. Furthermore, lixisenatide was not associated with a higher rate of serious adverse events [72]. In the LEADER trial, 9340 patients T2DM patients with high cardiovascular risk were randomized to receive liraglutide or placebo. Their findings showed that mortality outcome from cardiovascular causes was significantly lower in the liraglutide group compared to placebo (13% vs. 14.9%; P<0.001). However, gastrointestinal adverse events were reported in the treatment group [61]. 

Marso et al., in their trial, reported that out of 2735 T2DM with established cardiovascular disease, mortality due to cardiovascular causes was reported in 6.6% in the semaglutide group compared to 8.9% in placebo [68]. In the EXSCEL trial, 14,752 T2DM patients were enrolled with 73.1% having a CVD. The findings showed non-inferiority of the exenatide group in terms of death due to CVD causes compared to placebo [69]. GLP-1 RAs have demonstrated numerous positive impacts on patients' quality of life. Clinical data show that GLP-1 RAs, used as monotherapy or in combination with other glucose-lowering medications, are either superior or non-inferior in reducing HbA1c compared to other glucose-lowering medications [67]. Obesity, a chronic condition associated with T2DM, significantly impacts an individual's physical and psychological quality of life, as well as healthcare costs [74]. It is linked to multiple comorbidities including T2DM, hypertension, obstructive sleep apnea, and osteoarthritis-related joint pain [74,75]. A key feature of GLP-1 RAs is their superior ability to reduce weight. The low dosing frequency and ease of administration make GLP-1 RAs preferred by many diabetic patients. Furthermore, the rapid effects of this class on weight and HbA1c enhance patient adherence and reduce the likelihood of non-compliance [76].

Recommendations

Based on the evidence presented in this review, several recommendations can be made for the use of GLP-1 RAs in the management of type 2 diabetes and cardiovascular risk. Firstly, healthcare providers should consider GLP-1 RAs as a preferred treatment option for patients with T2DM, especially those with established cardiovascular disease or at high risk for cardiovascular events. Secondly, the choice of specific GLP-1 RA should be individualized based on patient characteristics, preferences, and cardiovascular risk profile. Thirdly, close monitoring of patients initiated on GLP-1 RAs is essential, particularly for gastrointestinal side effects in the early stages of treatment. Finally, further research is needed to explore the long-term cardiovascular benefits and potential risks of newer GLP-1 RAs and combination therapies.

Strengths

This review offers several notable strengths. It delivers a comprehensive overview of the current evidence on GLP-1 RAs, encompassing their mechanisms of action, glycemic control efficacy, and cardiovascular benefits. The incorporation of multiple large-scale cardiovascular outcome trials enables a robust evaluation of the cardiovascular safety and potential advantages of various GLP-1 RAs. Moreover, the review examines patient-centered outcomes - such as quality of life and treatment adherence - which are vital for the long-term management of diabetes. The comparative analysis of different GLP-1 RAs yields valuable insights for clinical decision-making and tailored treatment strategies.

Limitations

Despite its strengths, this review has limitations. First, the long-term effects of GLP-1 RAs beyond the duration of the included clinical trials remain uncertain. Second, the review's primary focus on cardiovascular outcomes may not fully capture other potential long-term effects or rare adverse events. Future reviews should address these limitations and incorporate emerging data on newer GLP-1 RAs and combination therapies.

## Conclusions

In conclusion, GLP-1 RAs have adequate safety and efficacy in the management of DM. GLP-1 RAs not only offer glycemic control in DM but also offer cardiovascular protection. These medications effectively lower HbA1c levels and contribute to weight loss, which are critical factors in the overall management of diabetes. The cardiovascular advantages of GLP-1 RAs are particularly important in patients with DM as they are at increased risk of CVD. Tirzepatide, mazdutide, cagrisema, and semaglutide are effective GLP-1 RAs for lowering HbA1c levels. Despite the efficacy of GLP-1 RAs in the management of DM, their use is often associated with some adverse events. In most cases, gastrointestinal complications such as nausea, and diarrhea are major concerns. However, some pancreatic and thyroid safety concerns have also been reported in the literature. Most of the adverse effects are dose-dependent and can be easily managed by adjusting the dose of GLP-1 RAs. Overall, GLP-1 RAs offer a valuable therapeutic option that addresses both glycemic control and cardiovascular health in DM patients.
